# The Human Corrosive Sublimate Tablet

**Published:** 1915-03

**Authors:** 


### The Human Corrosive Sublimate Tablet.

Cases of accidental and possibly criminal poisoning by antiseptic tablets have become relatively numerous and have called forth restrictive legislation. The intention of the bi-chlorid tablet is good: to destroy harmful germs, bacterial, protozoic and otherwise, as well as harmful vermin. Carelessly used—and everything kept on hand in accessible places is liable to be carelessly used—it has also destroyed human life and that of the higher animals. It has also been deliberately used to destroy human life in its initial, microorganismic form and, probably, this has been the ultimate purpose in the cases in which accidental deaths have occurred.

It is unnecessary to discuss at length the need of caution in the employment, especially by the laity, of highly toxic drugs. We wish to call attention to the possible danger from sociologic disinfectants. Our modern civilization has developed a considerable leisure class, a still larger class whose time is not fully occupied in the sordid and selfish attempt to secure sufficient food, clothing, shelter and other necessities for themselves while by formal and indirect education, it has made available for altruistic energies of a public nature, many individuals whose life would, under earlier conditions, have been industrial, domestic or occupied with personal amusement. Thus the country has widely distributed throughout its homes, social, economic and political organizations, an enormous number of individuals whose dominant energy is to diffuse an antiseptic action on the germs of domestic, social, industrial and political disease. Like the literal bichlorid tablet, these human antiseptic agencies are liable to be used carelessly, and ignorantly. They are liable to kill, not only sociologic disease germs but useful forces and they are especially liable to reduce the fecundity of various social and economic movements, not always perhaps representing ideal conceptions but still able to develop into useful units of human activity.

